# The effectiveness of asking behaviors among 9–11 year-old children in increasing home availability and children’s intake of fruit and vegetables: results from the Squire’s Quest II self-regulation game intervention

**DOI:** 10.1186/s12966-017-0506-y

**Published:** 2017-04-21

**Authors:** Ann DeSmet, Yan Liu, Ilse De Bourdeaudhuij, Tom Baranowski, Debbe Thompson

**Affiliations:** 1 Department of Movement and Sport Sciences, Faculty of Medicine and Health Sciences, Ghent University, Watersportlaan 2, Ghent, Belgium; 2 USDA/ARS Children’s Nutrition Research Center, Department of Pediatrics, Baylor College of Medicine, 1100 Bates St. Houston, TX 77030 Houston, USA

**Keywords:** Fruit, vegetable, diet, home availability, consumption, child, serious game, asking behaviors, intervention, goal setting

## Abstract

**Background:**

Home environment has an important influence on children’s fruit and vegetable (FV) consumption, but children may in turn also impact their home FV environment, e.g. by asking for FV. The Squire’s Quest II serious game intervention aimed to increase asking behaviors to improve home FV availability and children’s FV intake. This study’s aims were to assess: 1) did asking behaviors at baseline predict home FV availability at baseline (T0) (RQ1); 2) were asking behaviors and home FV availability influenced by the intervention (RQ2); 3) did increases in asking behaviors predict increased home FV availability (RQ3); and 4) did increases in asking behaviors and increases in home FV availability mediate increases in FV intake among children (RQ4)?

**Methods:**

This is a secondary analysis of a study using a randomized controlled trial, with 4 groups (each *n* = 100 child–parent dyads). All groups were analyzed together for this paper since groups did not vary on components relevant to our analysis. All children and parents (*n* = 400 dyads) received a self-regulation serious game intervention and parent material. The intervention ran for three months. Measurements were taken at baseline, immediately after intervention and at 3-month follow-up. Asking behavior and home FV availability were measured using questionnaires; child FV intake was measured using 24-h dietary recalls. ANCOVA methods (research question 1), linear mixed-effect models (research question 2), and Structural Equation Modeling (research questions 3 and 4) were used.

**Results:**

Baseline child asking behaviors predicted baseline home FV availability. The intervention increased child asking behaviors and home FV availability. Increases in child asking behaviors, however, did not predict increased home FV availability. Increased child asking behaviors and home FV availability also did not mediate the increases in child FV intake.

**Conclusions:**

Children influence their home FV environment through their asking behaviors, which can be enhanced via a serious game intervention. The obtained increases in asking behavior were, however, insufficient to affect home FV availability or intake. Other factors, such as child preferences, sample characteristics, intervention duration and parental direct involvement may play a role and warrant examination in future research.

**Trials Registration:**

ClinicalTrials.gov NCT01004094. Date registered 10/28/2009

**Electronic supplementary material:**

The online version of this article (doi:10.1186/s12966-017-0506-y) contains supplementary material, which is available to authorized users.

## Background

Eating sufficient fruit and vegetables (FV) is important for children’s health [1–3]. In many developed countries, child FV intake is below the recommended guidelines [4–7]. Children 9–13 years of age should eat at least 1.5-3 cups of V and 1.5-2 cups of F per day, whereas children in the United States on average have one cup of V and just over one cup of F per day [8]. Children’s FV intake is strongly influenced by their home environment [9–14], while children, conversely, may influence their home environment to facilitate a healthy diet [15, 16]. Most research on parent–child influences has focused on the unilateral influence of parents on children’s FV consumption [17, 18], or were cross-sectional in nature, not providing an opportunity to establish directional influences [19]. Some studies showed an increase in home FV availability after interventions that increased children’s asking for FV [20, 21]. Asking behaviors were defined as children asking their parents to make FV available at home or when eating out [20]. Reciprocal influences have been documented between children and parents among asking behavior and food choices [22].

Few studies have examined the role of children’s asking for healthy food items on the home food environment [20–22]. Families may be more open to children’s influences due to recent democratic models of family communication [23]. The marketing literature indicated that children’s asking behavior influenced parents’ food purchases [24]. Children were especially successful influencing ideas and decisions for purchases of sweets, FV, snacks, breakfast and easy-to-prepare meal purchases [23]. Children used persuasive strategies (e.g. expressing opinions, preferences, begging), bargaining (e.g. offering deals, such as cleaning up their room in return for the requested purchase) or emotional strategies (e.g. silent treatment, pestering) to influence their parents’ food purchases [25]. Children mainly requested unhealthy food items when shopping with their parents, to which parents often reluctantly conceded [26]. Encouraging children to request healthy items may increase the family’s healthy food purchases [26] and thus home FV availability.

Home FV availability is often studied in relation to other concepts such as FV accessibility, children’s awareness of FV availability and children’s preferences for FV. Some studies also combine the measurement of home FV availability and accessibility in one concept, making it difficult to distinguish their singular influences [27]. In this study, home FV availability was defined as whether FV were present in the home environment, e.g. in the fridge. Accessibility was defined as whether FV were accessible to children, e.g. FV in easy-to-reach locations and ready-to-consume forms [27]. Whenever studies have used items on both availability and accessibility, it will be reported as such. This study investigated home FV availability, but not accessibility.

Children without FV available at home were less likely to meet FV intake recommendations [13, 19, 27–31]. Meal-time FV availability predicted FV consumption one year later [32]. Increased home FV availability and accessibility predicted a sustained increase in FV intake at 18-month follow-up [33]. Home FV availability may be easy to manipulate [27]. Among preschoolers positive, but non-significant changes in home FV availability were found [34], whereas among elementary school children significant changes in home F and/or V availability were obtained [35]. Home FV availability may increase visual cues and exposure, which may impact children’s preferences for eating FV [28]. Preference refers to a predisposition to like certain foods and have an aversion for other types of food, but this may be changed through continued exposure to a certain food and the social context in which it is offered [28]. Parents may make FV available at home, e.g. by buying them and storing them in the fridge, but this may go unnoticed by children, in which case children are unaware of this availability. Home availability was related to healthy food intake only if children were aware of their availability [29]. Awareness may be enhanced by children’s involvement in food preparation and shopping [29], e.g. by children’s asking to prepare certain recipes or putting items on the shopping list [20].

Squire’s Quest II (SQII), a serious game, was designed to increase children’s FV intake, promoting children’s asking behaviors to increase home FV availability [36]. A serious game can increase healthy lifestyles such as a healthy diet [37]. Games create possibilities to practice healthy lifestyles, change mediators, apply change procedures such as tailoring or goal-setting [36, 38], and may intrinsically motivate to play for a longer time [39, 40]. SQII included goal-setting, educational supplemental material for parents, and direct involvement of parents via the children (e.g. using asking behaviors and recipe preparations).

This study’s research questions (RQ) included: 1) did asking behaviors at baseline predict home FV availability at baseline (T0) (RQ1); 2) were asking behaviors and home FV availability influenced by the intervention (RQ2); 3) did increases in asking behaviors predict increased home FV availability (RQ3); and 4) did increases in asking behaviors and increases in home FV availability mediate increases in FV intake among children (RQ4). We hypothesized that a) child asking behaviors would correlate with home FV availability; b) asking behaviors and home FV availability would increase at T1 and T2; and c) these increases would mediate a change in FV intake among children at T1 and T2.

## Methods

### Study design and sample

The SQII study used a randomized controlled design with four groups who all received an intervention, setting goals to increase FV consumption, but varied in their use of implementation intentions (action, coping, coping and action, none) (total *n* = 400 parent/child dyads). The conditions only varied in the extent to which implementation intentions were created to consume another serving of FV. All groups received a self-regulation intervention and set goals to increase FV consumption. The control condition did not create implementation intentions, intervention group 1 created action plans, intervention group 2 created coping plans, and intervention group 3 created both action and coping plans. This paper is a secondary analysis of the randomized controlled trial, reported elsewhere [41]. Since all groups received the asking behavior and parental intervention components, results here are presented as repeated measures comparisons for all groups together. Families were recruited using a convenience sample of attendees at community events, people responding to flyers, and the volunteer database at the Children’s Nutrition Research Center (Houston, TX USA). Eligibility criteria were a child in 4^th^–5^th^ grade of elementary school, having home access to high speed Internet, and a parent fluent in English or Spanish. This study was approved by the Baylor College of Medicine Institutional Review Board, written informed consent and child assent were obtained prior to participation in study activities.

The intervention was available for 3 months and played at home. Independent and dependent variables were assessed at baseline (T0), immediately post intervention (T1), and 3 months after the end of the intervention (T2). Data were collected between November 2009 and March 2011.

### Intervention

SQII is a serious game intervention to increase child FV intake. The intervention consisted of a 10-episode online videogame, set in the virtual Kingdom of Fivealot which featured an action adventure theme. Children were squires who had to overcome challenges (such as consuming FV and using recipes in real life) to become knights to help the King and Queen protect the kingdom. Children were eligible to play the next episode after pre-set interval; an eligibility reminder was sent [36].

#### Asking behaviors

Children were encouraged to ask their parents to add their favorite FV on the menu; make FV recipes together; add their favorite FV to the shopping list or buy these; be able to join their parents when grocery shopping for FV; and have FV in easy-to-reach places. Children were taught the PART acronym to ask or negotiate for FV in a manner most likely to be effective: “be *P*olite, *A*sk with confidence, be *R*easonable, good *T*iming, be *PART* of the solution”. Through modeling and dialogue, game characters demonstrated why it was important to use these techniques, and provided examples of how to use them. At goal review in the next episode, children were asked if they had used the PART strategy, for which they received positive reinforcement from the wizard avatar.

#### Parent component

Parents received information via electronic newsletters and a website, which were updated every played episode, to accompany the appropriate game content. Newsletters contained the episode goals and tips for parents on how to support their child in meeting their goals, information needed to facilitate their child’s game play (e.g. difficult words in the game), healthy FV recipes that were easy to prepare, and suggestions for overcoming common problems families face when attempting to eat FV (e.g. cost and time barriers, fit with children’s preferences). The parent website provided practical tips on creating a home environment promoting a healthy diet, such as FV recipes, grocery shopping tips, fast healthy meal suggestions (e.g. veggie wrap, paella), and information on promoting family physical activity.

The intervention was effective in increasing FV intake in the ‘action group’ at immediate post-intervention measurement and at three-month follow-up, and in the ‘coping planning group’ at immediate post-intervention measurement alone. The intervention results also showed favorable energy density changes at follow-up compared to baseline, only in the ‘action group’ and ‘action and coping planning group’ [42]. Detailed results including CONSORT diagram were provided elsewhere [41, 42].

### Measures

#### Children’s asking behavior (child-reported)

Children were asked in an online survey how often in the last two weeks they asked their parents to have FV available; have these in easy to reach places; to shop for or buy FV; to let them add FV to the shopping list; to ask for FV with a meal dining out; or make FV recipes together (e.g. “In the last two weeks, have you asked your parent or guardian to… have fruit or vegetables at home for breakfast?”, full scale provided in Additional file 1). A 9-item scale was used to record this behavior, using the response options ‘yes’ (2), ‘I don’t have to ask’ (1), ‘no’ (0) [20]. “I don’t have to ask” was included to distinguish those children who lived in homes where parents already provided FV from children who improved asking behaviors after participating in the intervention. An index summed responses to all items, resulting in a score ranging from 0–18. Cronbach α was α = 0.79 at baseline measurement (T0); α = 0.77 at immediate post-intervention measurement (T1); and α = 0.85 at follow-up (T2).

#### Home availability of fruit and vegetables (parent-reported)

A 40-item scale asked about home availability of 40 types of FV for the last two weeks in an online survey [43] (e.g. “In the last two weeks, have you had these vegetables, fruit, 100% fruit juices in your home? carrots, bananas,…). This scale was previously validated against observation of food in the home [44] and shown to be related to intake in another study [45]. This list was based on the types of FV most commonly consumed by a nationally representative sample of US children. Home availability used response categories ‘yes’ (2), ‘not sure’ (1), ‘no’ (0), resulting in a score ranging from 0–80. Cronbach α was α = 0.77 for baseline measurement (T0); α = 0.82 at immediate post-intervention measurement (T1); and α = 0.71 at follow-up (T2).

#### Child FV intake (child-reported)

Child FV intake was assessed using 24-h dietary recalls from the child using the premier NDS-R computerized interview (Nutrient Data System for Research, NDSR-2009) [46] on three unannounced occasions for each data collection period (T0, T1, T2), by trained staff. The values were an average across the three days thus lending some reliability as an indicator of habitual intake over a two week period of time. Self-report measures are known to contain a substantial amount of error, but for a variety of reasons 24hdr using NDS-R are considered by many to be the most accurate, and thereby preferred. At each occasion, recalls comprised two weekdays and one weekend day recall. The dietary recalls were analyzed for FV servings [36].

### Analysis

Analyses of Covariance (ANCOVA) were used to examine whether child asking behaviors at baseline correlated with home FV availability at baseline (RQ1), controlling for possible confounders such as child’s gender, race/ethnicity, social desirability measured by the lie scale of the Revised Children’s Manifest Anxiety Scale [47], and parent’s education and age. A linear mixed-effect model with maximum likelihood estimation (MLE) was applied to determine whether and how much the outcomes of child asking behaviors and home FV availability changed after the intervention (RQ2). Since all conditions received the goal-setting and parental component intervention, time effects were the main focus of interest. Analyses, however, first assessed significance of group x time interaction effects. The dependent variables were child asking behaviors and home FV availability. A four-level between-subject factor (intervention groups) and a three-level within-subject factor (time: T0, T1, and T2) were treated as independent fixed factors, where subjects were treated as a random factor. The models were adjusted for child’s gender, race/ethnicity, social desirability, and parent’s education and age. Post hoc analyses (Tukey’s HSD) were conducted to compare differences between specific measurement times (T0, T1, T2). Statistical Analysis Software (SAS version 9.4) was used for these analyses. Minor differences in results can be noted with the main intervention outcome paper [41] due to a slightly different sample size and other analytical methods.

Structural equation modeling (SEM) was used to test RQ3 and RQ4 using Mplus 6.12. SEM models examine the predictive association of two variables over time, each controlling for the effects at earlier time points. Cole’s guidelines for conducting SEM were followed [48]. To test the hypothesized models and to explore for possible reciprocal and stationary effects, analyses were conducted to: 1) assess measurement invariance; 2) test the overall proposed structural model; 3) test for mediation effects; and 4) examine reciprocal and stationary effects. Models for RQ 3 and 4 controlled for baseline measures, child’s age and gender. Because there were four different experimental conditions, a four-group measurement model and structural equation model were constructed to establish measurement comparability. Since measurement equivalence across conditions and no group-specific differences were found, the four conditions were combined for all subsequent analyses.

Fit of all models was evaluated with a minimum fit function chi-square test (*χ* ^2^) and other approximate indicators, including the root mean square error of approximation (RMSEA), the Tucker-Lewis Index (TLI) and the comparative fit index (CFI) using the following criteria: *χ* ^2^ (not significant, p-value >0.05), RMSEA (criterion ≤0.07), and TLI and CFI (criterion ≥0.95) [49].

## Results

### Descriptive results

Four-hundred children participated at baseline measurement (T0) (52.5% girls). Of the 400 children randomized to the conditions, 392 completed the immediate post-intervention measurement (T1) (98.0%) and 387 completed the follow-up measurement (T2) (96.8%). There were no significant differences in socio-demographic characteristics between completers (*n* = 387) and non-completers (*n* = 13). The sample was multi-ethnic (36.8% Caucasian, 27.0% Hispanic, 26.3% African American, 10.0% other racial/ethnic groups). For each child, one parent participated (96.3% mothers, 55.5% 40 years of age or older). The majority of parents were highly educated (31.5% college graduate, 36.0% post-graduate), and married (77.5%). There were no significant differences at baseline among the participants in the four experimental conditions regarding child’s gender, child’s or parent’s ethnicity, parent’s age, educational level or marital status. The mean child FV consumption was 2.1 daily servings at baseline (T0, SD = 1.3), 2.6 servings immediately post-intervention (T1, SD = 1.7), and 2.4 servings daily at follow-up measurement (T2, SD = 1.5) (Table 1).

**Table 1:** Averages for dependent and independent variables across measurement times

	M (SD)		
	Baseline, T0	Immediate post-intervention measurement, T1	Follow-up measurement, T2
	Average (SD)		
Child FV intake^a^	2.1 (1.3)	2.6 (1.7)	2.4 (1.5)
		Cohen’s d_T1-T0_ = 0.36	Cohen’s d_T2-T0_ = 0.23
Child FV asking behavior^b^	9.9 (4.2)	11.8 (4.0)	11.0 (4.2)
		Cohen’s d_T1-T0_ = 0.47	Cohen’s d_T2-T0_ = 0.26
Home FV availability^c^	40.1 (10.7)	47.0 (10.2)	44.9 (9.6)
		Cohen’s d_T1-T0_ = 0.66	Cohen’s d_T2-T0_ = 0.47

Note. Measurement units^a^ Servings per day; ^b^Number of asking behaviors for FV (1 behavior = 2 points); ^c^Number of FV available at home (1 F/V = 2 points)

### Prediction of home FV availability by child’s asking behaviors (T0, baseline)

Asking behaviors at baseline were significantly positively associated with baseline home FV availability (unstandardized β coefficient = 0.36, SE = 0.14, F_(1, 319)_ = 6.53, *p* < .05), suggesting that for one unit increase in asking behaviors, on average home FV availability increased by 0.4 points. Adjusted for potential confounders, the explained variance of asking behaviors at baseline associated with home FV availability at baseline was 4.7%. These findings support the first research hypothesis, that child asking behaviors correlated with home FV availability.

### Changes in child asking behaviors and home FV availability after the intervention

**Fig. 1:**
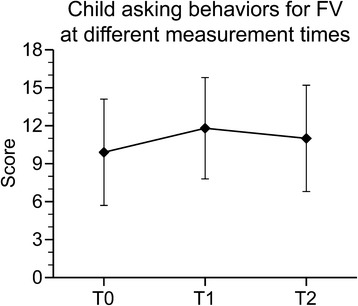
Child asking behaviors at different measurement times. Model adjusted for child’s gender, race, social desirability and parent’s education and age

There were no statistically significant group × time interaction effects on child asking behavior or home FV availability; therefore, these interaction terms were removed from the final models. There were no statistically significant main intervention group effects on any dependent variable (all F < 1.55, *p* > 0.05). Significant time effects on child asking behaviors (F_(2,650)_ = 32.53, *p* < 0.0001) were observed (Fig. 1). Post hoc analyses showed that child asking behaviors were significantly higher immediate post-intervention (T1) compared to baseline (T0) (t = 8.12, *p* < .0001), and that child asking behaviors were significantly lower at follow-up measurement (T2) compared to immediate post-intervention measurement (T1) (t = 4.68, *p* < .0001), but still significantly higher when compared to baseline (t = −3.44, *p* = .0006).

Children’s mean FV asking behavior had an average of 9.9 points at baseline (SD = 4.2), and increased by 1.9 points more asking behaviors at T1 compared to baseline, and by 1.1 asking behaviors at T2 compared to baseline.

**Fig. 2:**
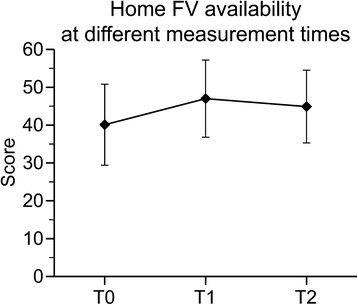
Home FV availability at different measurement times. Model adjusted for child’s gender, race, social desirability and parent’s education and age

Significant time effects were also observed for home FV availability (F_(2,650)_ = 101.59, *p* < 0.0001) (Fig. 2). Compared to baseline, mean home FV availability significantly increased (t = 14.46, *p* < .0001) by 6.9 points (2 points representing one type of F or V) at measurement immediately after the intervention (T1), and by 4.7 points at measurement three months after the intervention ended (T2) compared to baseline (t = 9.50, *p* < .0001). Although significantly reduced at T2 compared to T1 (t = −4.96, *p* < .0001), home FV availability was still significantly higher at T2 than at baseline (T0). These findings support our second hypothesis that asking behavior and home FV availability had increased after the intervention, although the changes immediately after the intervention were not fully maintained at follow-up.

### Prediction of increased home FV availability by increased asking behaviors

Measurement invariance using confirmatory factor analysis (CFA) was conducted to test whether the latent constructs of child asking behavior and home FV availability were comparably assessed across three measurements (T0, T1, and T2). The configured invariance (freely estimated model constructed) demonstrated good fit (*χ* ^2^_(294,341)_ = 353.8, *p* = 0.294; RMSEA = 0.024; CFI = 0 .97; TLI = 0.96), suggesting that the pattern of free and fixed parameters was equivalent across measurement occasions. Next, the indicator factor loadings across occasions were tested using the metric invariance (weak factorial invariance) model. All factor loadings were constrained to be equal across time. The model had acceptable fit (*χ* ^2^_(310,341)_ = 392.6, *p* = 0.012; RMSEA = 0.028; CFI = 0.95; TLI = 0.95). No significant changes in model fit were observed after comparing to the configured invariance model (∆*χ* ^2^_(2)_ = 0.25 *p* = 0.884). Finally, equality of the indicator intercepts across time using scalar invariance (strong factorial invariance) was tested. The model fit was acceptable (*χ* ^2^_(322, 341)_ = 418.39, *p* = 0.0002; RMSEA = 0.030; CFI = 0.95; TLI = 0.95). No significant changes in model fit were observed compared to the metric invariance model (∆*χ* ^2^_(2)_ = 4.68, *p* = 0.096). Taken together, these tests indicated that measurement invariance was obtained across time periods when the constructs included child asking behavior and home FV availability in the model.

**Fig. 3:**
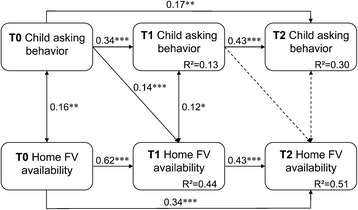
Autoregressive cross-lagged model of child asking behavior and home FV availability. Straight single arrows indicate the causal paths modeled, while the straight double arrows between variables represent a correlation. Numbers next to the paths show standardized path coefficients; bold face coefficients indicate statistically significant *p* < 0.05, while dotted lines are used for paths with *p* > 0.05. R-squared coefficients, a proportion of variance accounted for by exogenous variables, are displayed. *p < .05, **p < 0.01, ***p < .0001

The conceptual framework and results are shown in Fig. 3. The fit of the autoregressive longitudinal path model was adequate (*χ* ^2^_(8,341)_ = 14.01, *p* = 0.081; CFI = 0.99; TLI = 0.97; RMSEA = 0.047, 90% CI 0.00, 0.09). Child asking behaviors at T0 predicted home availability at T1, controlling for home availability at T0 (standardized coefficient = 0.14; *p* < 0.01; 95% CI 0.08, 0.21). Significant associations were observed between earlier and post-intervention measurements for asking behaviors and FV home availability (T0 to T1, T1 to T2, and T0 to T2; all coefficients *p* < 0.01). However, there was no significant lagged path from child asking behavior at T1 to home availability at T2. Non-significant paths were shown as dotted lines in Fig. 3. Paths not shown by a line were not hypothesized or investigated in our research questions. These findings lend only partial support for RQ3.

### Mediation effects of child asking behavior and home FV availability on child FV intake

**Fig. 4:**
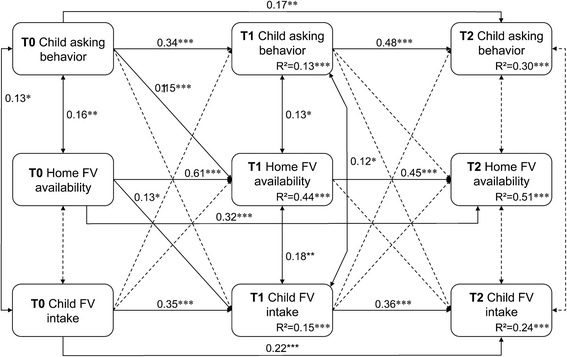
Autoregressive cross-lagged model among child asking, home FV availability, and child FV intake. Goodness-of-fit: *x*2_(10,341)_ = 13.88, *p*-value = 0.179; CFI = 0.99, TLI = 0.99, RMSEA = 0.034 (90% CI: 0.00, 0.07), SRMR = 0.028. Straight single arrows indicate the casual paths modeled, while the straight double arrows between variables represent a correlation. Numbers beside paths represent standardized path coefficients; bold face coefficients indicate statistically significant *p* < 0.05, while broken lines are used for paths with *p* > 0.05. The R-squares, a proportion of variance accounted for by exogenous variables, are displayed. *p < .05, **p < .01, ***p < 0.0001

A mediation path was examined to test whether home FV availability explained any influence of child asking behaviors on child FV intake (RQ4), controlling for child’s gender and race. Non-significant paths are shown in dotted lines in Fig. 4. The model goodness of fit was good (*χ* ^2^_(13,341)_ = 26.48, *p*-value = 0.015; CFI = 0.98; TLI = 0.95; RMSEA = 0.055, 90% CI 0.02, 0.09).

The conceptual framework and results are shown in Fig. 4. Paths not shown by a line were not hypothesized or investigated in our research questions. Non-significant paths are shown in dotted lines in Fig. 4.

Child asking behaviors at T0 predicted a significant lagged effect on home FV availability at T1 (standardized coefficient = 0.15, *p* < 0.01, 95% CI 0.07, 0.21), controlling for T0 home availability and T0 child FV intake. Home FV availability at T0 predicted child FV intake at T1 (standardized coefficient = 0.13, *p* < 0.01, 95% CI 0.06, 0.23), controlling for baseline child FV intake. However, the lagged effects of child asking behavior at T1 on home FV availability at T2, home availability at T1 on child FV consumption at T2, or child FV consumption on home FV availability at T2 were not significant. The hypothesized mediation was not significant.

## Discussion

This study investigated whether child asking behavior predicted home FV availability; whether both elements increased in a serious game self-regulation intervention; and whether increased asking behavior and increased home FV availability predicted a change in child FV intake.

The intervention resulted in small effect sizes on child FV intake and on child asking behaviors at immediate post-intervention measurement and at follow-up measurement; and in moderate effect sizes on home FV availability immediately post-intervention and small effect sizes at follow-up. While there is no comparison in the literature for the effects on asking behaviors, the effects obtained on child FV intake are in line with those obtained in FV interventions [50]. Although the effects are not close to the desired minimum of 5 servings a day, the public health community often states that even small average increases spread across a large population can have substantial benefits for some in that population [51].

### Home FV availability

Home FV availability at baseline predicted child FV intake immediate post-intervention (RQ2). Our results confirmed systematic review findings that home FV availability and accessibility influence children’s FV consumption [13, 19, 27–30]. Moreover home FV availability was increased by a serious game that directly involved children and provided information for parents, which was maintained at 3-month follow-up. Skill-teaching activities to increase children’s asking behaviors for FV influenced parents’ behavior in ensuring FV availability. This lends support to the reciprocal determinism principle that parents not only influence their children’s behavior, but that children in turn also influence their environment [52]. Our findings suggest that, intervening with children or parents alone may not be as effective as family-focused interventions for influencing FV.

Children’s asking behavior at baseline explained only a very small proportion of the variance in home FV availability at baseline (4.7%) (RQ1), suggesting other determinants may need to be considered to change home FV availability. The conceptual model for SQII assumed home FV availability was influenced by the level of asking behaviors, parental involvement (addressed via newsletters and recipe preparations), and child food preferences [36]. Other psychosocial or environmental determinants, such as social support for healthy eating [53], food security [53, 54], household composition characteristics and SES [53, 54], family meal patterns [53], parenting skills to promote FV, parental role modeling, lower perceived benefits of fast food, and child food preferences [54], may have, however, also played a role. Additional components to address in future interventions apart from these already included in SQII, may focus on environmental factors such as food security and psychosocial factors such as parenting skills, meal patterns, and increasing motivation to impact home FV availability.

The SQII intervention succeeded in increasing asking behaviors, but increased asking behaviors at first post-intervention measurement did not significantly predict home FV availability at follow-up. Both availability and asking behaviors decreased at follow-up yet remained higher than baseline. This suggests additional efforts are needed to maintain FV home availability and asking behaviors. Possibly, the 10-episode intervention was insufficient to create habitual asking behaviors and home FV availability. The e-mails containing the newsletters sent to parents may have served as a gentle reminder during the intervention even when their content was frequently not read. Using follow-up prompts is considered a behavioral change technique to enhance the maintenance of behavior change and was found to create large effects when combined with providing information on the behavior-health association [55]. Continuing these reminders or prompts for parents after the intervention has ended may contribute to a higher retention of the effect in future interventions. Text or e-mail messages for children could be tacked on to SQII after the game intervention has ended, to encourage children to keep up their asking behaviors. Further studies using SQII would be valuable which used qualitative measures to assess parent reactions to child FV asking behaviors from both the parent and child perspectives.

### Child FV intake

The increase in home FV availability achieved through the intervention (T1) also did not predict an increase in child FV intake at follow-up measurement. Several factors may explain this lack of effect, relating to 1) the size of the change, 2) other mediators that influence child FV intake, 3) the sample characteristics, or 4) type of parental involvement.

First, cross-sectional studies on predictive associations between home FV availability and child FV intake have shown significant [56], but also non-significant predictive associations [57, 58]. The cross-sectional design, however, does not permit predictive temporal associations between changes acquired through an intervention in FV home availability and child FV intake, as can be analyzed via intervention studies such as the SQII study. A previous intervention study showed that with an increase of one unit in home FV availability and accessibility (measured from −10 to +10), child FV intake increased by 0.14 units (ranging from 0–40). This could suggest home FV availability and accessibility needs to increase substantially to achieve a small change in child FV consumption [33]. The change in home FV availability obtained in our intervention study thus may have been too small to predict a change in child FV intake. Despite its relatively small impact on intake, home availability may, however, be a necessary condition to change FV intake. Among 12–13 year olds, a self-regulation computer-tailored intervention only changed vegetable consumption among children who always had vegetables available at home [59]. Thus, changing personal determinants may not be effective unless the home environment is already supportive of children’s V intake. Especially for vegetable intake, this is hypothesized to be important due to youngsters’ dependence on family meals for vegetable consumption [59]. In sum, although a large effect appears needed in home FV availability and accessibility to impact child FV intake, these changes in home environment may be necessary to facilitate effects from individual interventions.

Second, other mediators such as child preferences may play a role in child FV consumption. A cross-sectional study among 11–14 year olds from lower SES families showed that interaction, but not direct, effects of home FV availability and child taste preferences were predictive of child FV intake [60]. Taste preferences for FV showed a positive association with FV intake only when home FV availability was high. Home FV availability alone did not influence intake, suggesting preferences need to be addressed as well [60]. The SQII intervention aimed to improve preferences for FV by trying to increase exposure to FV as recommended in the literature [28]. The intervention did this by providing virtual kitchen recipes to children and recipes to parents for FV that are generally less preferred. Possibly, an extended intervention duration may have a greater impact on child FV intake. A meta-analysis also indicated that parental involvement and a longer duration were key success factors in weight-related interventions for children and adolescents [61]. Interventions ranged from 9 weeks to 4 years. Although no specific recommendation was provided for an optimal intervention duration, a linear relation was found between duration and effectiveness, showing our intervention duration was situated at the lower end of this range. A longer duration of the intervention may help establish habits in FV consumption, which appeared especially predictive of fruit intake [62].

Third, previous studies showed that home FV availability influenced elementary school children’s FV intake as a mediator in the relation between low parental educational level [63] or low nutritional knowledge [56] and FV intake. Increasing home FV availability may be especially important in families from low socio-economic background or with less nutritional knowledge. Lower SES groups indeed had lower V home availability than intermediate or high SES groups [63], and home food environment was the most important predictor of SES differences in healthy food consumption among 4^th^ graders [64]. With an average baseline value of 40 on the summed index of home FV availability (2 units = 1 type available), a ceiling effect may have occurred in this mostly higher SES sample in our study. Our intervention may thus have a larger effect on child FV intake in a sample of participants from a lower SES background.

Fourth, the intervention included indirect involvement of parents via children’s prompts, the website and newsletters, but this parental component was not widely used. Only 28% of parents reported reading more than half of the newsletters; 55% reported visiting the parent website 1–5 times; 32% 6–10 times; and 28% 11 or more times [41]. A systematic review suggested direct involvement of parents to be more effective in changing children’s dietary patterns than indirect involvement [65]. An option for direct parental in-game involvement may lie in providing a multiplayer serious game that are jointly played by children and parents. Positive outcomes have been reported of intergenerational multiplayer games, mostly applied to dyads of grandparents-grandchildren and outcomes on family interaction, communication, cognitive functioning, and learning [66]; and of multiplayer games co-played by parents and their children to ward off potential negative effects of media use and to improve parent–child relationships [67, 68]. Social facilitation, i.e. the presence of others, can be expected to increase the game-play effort and positive outcomes [69]. To our knowledge, no multiplayer serious game for parents and children promoting FV consumption and availability has been evaluated. Given positive findings in other areas, this could be a worthwhile direction for future intervention research.

#### Limitations and strengths

The study had some limitations. First, the findings are specific for the age group of this study (4th-5th graders) and may not transfer to younger or older children, or other cultural settings. Second, the sample mostly consisted of higher SES families, while home FV availability is more problematic among lower SES families. The intervention might have had a larger effect in lower SES populations than evidenced in this current study. Third, the study lacked a pure control condition that did not receive any intervention. Fourth, the asking scale was based on previous research but was not validated separately for this study. And lastly, our analyses used a sample of *n* = 387, which provided an acceptable level of power (84%) using the Monte Carlo simulations with bootstrap method [70, 71], to detect an effect size of d > 0.25. This power was sufficient to detect direct intervention effects on child asking behavior, child FV intake and home FV availability, but likely insufficient to detect mediated paths, which showed effect sizes much smaller than d = 0.25.

The study also had several strengths. The intervention was developed in an evidence- and theory-based manner, integrating theories not only to increase intention but also to translate positive intentions into action [36], and addressing both individual and environmental determinants of child FV intake. Although a longer intervention duration may yield higher effects, the intervention period was longer than commonly the case in serious game interventions [37]. This longer period allowed for more practice of skills and behaviors. The study was conducted in a rigorous methodological manner using validated scales. Lastly, the study findings were innovative in manipulating children’s asking behaviors and assessing outcomes on home FV availability as well as child FV intake. The study has led to novel insights and recommendations for future research.

## Conclusions

Home FV availability at baseline predicted child FV intake post-intervention. Child asking behaviors at baseline explained a small proportion of home FV availability at baseline, supporting reciprocal determinism principles that parents not only influence their children’s behavior, but that children in turn also influence their environment. The intervention succeeded in increasing child FV intake [41], home FV availability and child asking behaviors for FV immediately post-intervention, but somewhat decreased at follow-up. The intervention lead to more child asking behaviors and had a positive effect here. We found a significant mediation path from child asking behaviors at baseline to child FV intake after the intervention, mediated by home FV availability after the intervention. More child asking behavior after the intervention, however, did not lead to a sustained availability at follow-up and not to a sustained higher level of intake. The mediation path found earlier disappeared when investigating asking behaviors after the intervention and child FV intake and home FV availability at follow-up. Some hypotheses can be put forward to explain these findings. First, asking behaviors only had a small contribution to explaining the variance in home FV availability and other predictors may have influenced home FV availability at follow-up. Second, child asking behaviors and home FV availability dropped at follow-up. The increase in home FV availability at follow-up may no longer have been sufficiently large to predict an increase in child FV intake. This supports our recommendations to include follow-up reminders to maintain intervention effects in future developments of the intervention and to also address other predictors of home FV availability than child asking behaviors.

### Future directions

Suggestions for future research include extending intervention duration, incorporating post-intervention reminders and addressing other psychosocial and environmental factors. Future research should explore whether this intervention may yield larger effects among lower SES families and whether direct parental involvement can further improve the intervention’s outcomes. More intervention studies on home FV availability are needed that can shed light on the determinants and effects of increasing home FV availability on FV intake in different subpopulations, e.g. by directly comparing determinants and effects in different strata or by creating large enough samples across studies to enable studying these research questions in a meta-analysis.

## Additional file

Additional file 1: Asking behavior scale and home FV availability scale. (DOCX 13 kb)
