# Supplementary material for: The effectiveness of asking behaviors among 9–11 year-old children in increasing home availability and children’s intake of fruit and vegetables: results from the Squire’s Quest II self-regulation game intervention
Source: Int J Behav Nutr Phys Act. 2017 Apr 21;14:51. doi: 10.1186/s12966-017-0506-y (PMC5399846; doi:10.1186/s12966-017-0506-y)
Supplement: Additional file 1: — Asking behavior scale and home FV availability scale. (DOCX 13 kb) [file 12966_2017_506_MOESM1_ESM.docx]

**Additional file 1**

**Asking behavior scale (child-reported)**

Please click on the answer that shows if you have asked your parent or guardian these questions in the last 2 weeks.
(response options = yes, no, I don’t have to ask)

In the last two weeks, have you asked your parent or guardian to:
1. Have fruit or vegetables at home for breakfast?
2. Have fruit or vegetables at home for snacks?
3. Have fruit or vegetables at home for dinner?
4. Go with them grocery shopping for fruit and vegetables?
5. Go to a restaurant or fast food place and ask for fruit or vegetables with your meal?
6. Buy fruit or vegetables?
7. Have fruit or vegetables on the counter or in easy to reach places?
8. Let you add the fruit and vegetables you want to the grocery shopping list?
9. Make fruit and vegetable recipes with you?

**Home FV availability scale (parent-reported)**

In the last 2 weeks, have you had these vegetables in your home

(response options = yes, no, not sure)

1. Carrots
2. Celery
3. Greens (like collards, mustard greens, or spinach)
4. French fries
5. Potato salad
6. Other white potatoes
7. Corn
8. Green peas (English peas)
9. Tomatoes
10. Broccoli
11. Lettuce
12. Green beans
13. Cucumbers
14. Jalapenos
15. Salsa, pico de gallo
16. Bell peppers (green, yellow, orange, red)
17. Cooked beans (like pinto, black-eyed peas, red beans, pork & beans)
18. Sweet potatoes
19. Cabbage
20. Okra

In the last 2 weeks, have you had these 100% fruit juices or fruit in your home

(response options = yes, no, not sure)

1. 100% orange juice
2. 100% apple juice
3. Other 100% juices
4. Bananas
5. Apples
6. Cantaloupe or musk melon
7. Grapes
8. Oranges
9. Pears
10. Plums
11. Kiwi
12. Strawberries
13. Pineapple
14. Grapefruit
15. Fruit cocktail
16. Applesauce
17. Watermelon
18. Raisins
19. Dried fruit
20. Peaches
